# Stable isotope tempestology of tropical cyclones across the North Atlantic and Eastern Pacific Ocean basins

**DOI:** 10.1111/nyas.15274

**Published:** 2024-12-19

**Authors:** Ricardo Sánchez‐Murillo, Dimitris A. Herrera, Kegan K. Farrick, Germain Esquivel‐Hernández, Rolando Sánchez‐Gutiérrez, Javier Barberena‐Moncada, Jorge Guatemala‐Herrera, Yelba Flores‐Meza, Roberto Cerón‐Pineda, Laura Gil‐Urrutia, Jorge Cardona‐Hernández, Tania Peña‐Paz, Junior O. Hernández‐Ortiz, Wendy Harrison‐Smith, Geoffrey Marshall, Aurel Persoiu, Juan Pérez‐Quezadas, Miguel Mejía‐González, Luis González‐Hita, Marcia Barrera de Calderón, Alejandro García‐Moya, Debora Hernández, Kristen Welsh, Rene M. Price, Diego A. Riveros‐Iregui, Ny Riavo G. Voarintsoa, Joshua C. Bregy, Minerva Sánchez‐Llull, Carlos Alonso‐Hernández, Saúl Santos‐García, Ana M. Durán‐Quesada, Christian Birkel, Jan Boll, Kim M. Cobb, Adrián F. Obando‐Amador, Ingrid M. Vargas‐Azofeifa, Doerthe Tetzlaff, Chris Soulsby, Sylvia G. Dee

**Affiliations:** ^1^ Department of Earth and Environmental Sciences University of Texas at Arlington Arlington Texas USA; ^2^ Department of Geography & Sustainability University of Tennessee Knoxville Tennessee USA; ^3^ Instituto Geográfico Universitario Universidad Autónoma de Santo Domingo Santo Domingo Dominican Republic; ^4^ Department of Geography The University of the West Indies St. Augustine Trinidad and Tobago; ^5^ Stable Isotopes Research Group & Water Resources Management Laboratory, School of Chemistry Universidad Nacional Heredia Costa Rica; ^6^ Centro para la Investigación en Recursos Acuáticos de Nicaragua (CIRA) Universidad Nacional Autónoma de Nicaragua Managua Nicaragua; ^7^ Ministerio de Medio Ambiente y Recursos Naturales San Salvador El Salvador; ^8^ Facultad Ciencias de la Tierra y la Conservación Universidad Nacional de Agricultura Catacamas Honduras; ^9^ Centro Experimental y de Innovación del Recurso Hídrico (CEIRH) Universidad Nacional Autónoma de Honduras Tegucigalpa Honduras; ^10^ National Water Commission Kingston Jamaica; ^11^ Water Resources Authority of Jamaica Kingston Jamaica; ^12^ Emil Racovita Institute of Speleology Romanian Academy Cluj‐Napoca Romania; ^13^ Stable Isotope Laboratory Stefan cel Mare University, Suceava Suceava Romania; ^14^ Instituto de Geología Universidad Nacional Autónoma de México Ciudad de México Mexico; ^15^ Department of Hydrology Mexican Institute of Water Technology Jiutepec Mexico; ^16^ Facultad de Ciencias Agronómicas Universidad de El Salvador San Salvador El Salvador; ^17^ Departamento de Estudios de la Contaminación Ambiental Centro de Estudios Ambientales de Cienfuegos Cienfuegos Cuba; ^18^ Centro de Aplicaciones Tecnológicas y Desarrollo Nuclear La Habana Cuba; ^19^ Geosciences Department Oberlin College and Conservatory Oberlin Ohio USA; ^20^ Small Island Sustainability Programme University of The Bahamas Nassau Bahamas; ^21^ Department of Earth and Environment Florida International University Miami Florida USA; ^22^ Department of Geography and Environment University of North Carolina at Chapel Hill Chapel Hill North Carolina USA; ^23^ Department of Earth and Atmospheric Sciences University of Houston Houston Texas USA; ^24^ Department of Environmental Engineering and Earth Sciences Clemson University Clemson South Carolina USA; ^25^ Department of Management and Environmental Engineering Centro de Estudios Ambientales de Cienfuegos Cienfuegos Cuba; ^26^ International Atomic Energy Agency Monaco Monaco; ^27^ Unidad de Hidrología, Dirección General de Infraestructura Nacional Secretaría de Infraestructura y Transporte Tegucigalpa Honduras; ^28^ School of Physics & Environmental Pollution Research Center University of Costa Rica San José Costa Rica; ^29^ Department of Geography, Water, and Global Change Observatory University of Costa Rica San José Costa Rica; ^30^ Department of Civil and Environmental Engineering Washington State University Pullman Washington USA; ^31^ Department of Earth, Environmental, and Planetary Sciences Brown University Providence Rhode Island USA; ^32^ Instituto Costarricense de Acueductos y Alcantarillados San José Costa Rica; ^33^ Escuela Centroamericana de Geología Universidad de Costa Rica San José Costa Rica; ^34^ IGB Leibniz IGB Leibniz Institute of Freshwater Ecology & Inland Fisheries, Humboldt University Berlin Berlin Germany; ^35^ School of Geosciences University of Aberdeen Scotland UK; ^36^ Department of Earth, Environmental, and Planetary Sciences Rice University Houston Texas USA

**Keywords:** climate change, extreme events, oxygen and hydrogen isotopes, paleoclimate, precipitation contribution, surface water and groundwater, water management

## Abstract

Tropical cyclones (TCs) are one of the major natural hazards to island and coastal communities and ecosystems. However, isotopic compositions of TC‐derived precipitation (P) in surface water (SW) and groundwater (GW) reservoirs are still lacking. We tested the three main assumptions of the isotope storm “spike” hypothesis (sudden spikes in isotopic ratios). Our database covers 40 TCs and is divided into recent (*N* = 778; 2012–2023) and archived (*N* = 236; 1984–1995) rainfall isotope observations and SW/GW isotope monitoring (*N* = 6013; 2014–2023). Seasonal rainfall contribution from TCs ranged from less than 1% to over 54% (4% on average) between 1984 and 2023. Mean δ^18^O compositions across TCs domains were significantly lower than the regional (noncyclonic) δ^18^O mean (−5.24 ± 4.27‰): maritime (−6.29 ± 3.28‰), coastal (−7.78 ± 4.28‰), and inland (−9.80 ± 5.18‰) values. Coastal and maritime TC convection resulted in large rainfall amounts with high isotope compositions. This could bias past climate reconstructions toward unrealistic drier conditions. Significant δ^18^O and *d*‐excess differences were found between storm intensities. P/SW and P/GW isotope ratios revealed the rapid propagation of TC excursions in freshwater systems. Our findings highlight the potential of TC isotope observations for diagnosing intensity and frequency in paleoproxies beyond idealized TC models.

## Graphical abstract

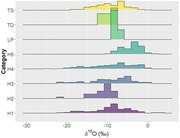

## INTRODUCTION

The North Atlantic, Caribbean Sea, Gulf of Mexico, and Eastern Pacific Ocean basins have been identified as hotspots for their sensitivity to climate change.^1^, ^2^, ^3^, ^4^ Across these regions, tropical cyclones (TCs) are one of the major natural hazards of fragile coastal ecosystems and low‐income communities.^5^, ^6^, ^7^, ^8^ More intense and frequent TCs coupled with strong interannual rainfall variability and severe droughts often result in extensive ecological disruptions, economic losses, and fatalities.^9^, ^10^, ^11^, ^12^, ^13^ These hydroclimate extremes are also among the main drivers for large human migrations into North America,^14^, ^15^, ^16^ with 2.5 million encounters in the southwest land border in 2023.^17^

Climate models suggest that TCs will likely intensify as a result of climate change, with a 10%–15% increase in the frequency of major hurricanes (categories 3–5) by 2050.^18^, ^19^ Uncertainties and constraints in TC projections under a warmer climate could be reduced and improved by understanding how TC frequency, intensity, and spatiotemporal distribution have varied since the start of the instrumentation era, approximately 1851 CE.^20^, ^21^, ^22^, ^23^, ^24^, ^25^ Despite their effects as natural hazards and their associated threats, TCs can benefit groundwater (GW) recharge and alleviate drought depending on interannual rainfall deficits and antecedent soil moisture conditions.^26^, ^27^, ^28^, ^29^, ^30^

Several recent studies document the utility of stable water isotope measurements for capturing TC frequency and storm microphysical characteristics.^31^, ^32^, ^33^ Climate models with embedded water isotopes (^18^O and ^2^H) are used in the interpretation of paleoclimate proxies (e.g., caves, tree rings, and sediments),^34^ which may help to translate the effects of frequency and intensity of TCs into improved future risk management plans and ecohydrological assessments.^22^, ^23^, ^35^, ^36^, ^37^, ^38^ In terms of paleoclimate, for example, subannually resolved speleothem (i.e., mineral deposits formed from GW within caves) records from Belize indicate TC activity in the western Caribbean Sea peaked in the mid‐1600s before declining throughout the latter half of the Little Ice Age and into the late 20th century due to a northward migration in storm tracks.^39^ Recently, a 473‐year‐long tree‐ring proxy record was reconstructed in southern Mississippi, United States, by using longleaf pine from excavated coffins, historical houses, remnant stumps, and living trees.^23^ They found that TC precipitation significantly declined in the 2 years following large Northern Hemisphere volcanic eruptions and was influenced by the behavior of the North Atlantic subtropical high‐pressure system. This is consistent with the rapid effects of simulated volcanic eruptions on the intensity of TCs.^40^

Similarly, isotopic measurements in the α‐cellulose of tree rings have been used to reconstruct TC activity^41^; however, inaccuracies in TC frequency estimations may arise due to false positives (i.e., ^18^O‐depletion in years without a TC) and false negatives (i.e., no ^18^O‐depletion despite the occurrence of a TC).^41^, ^42^ This issue may be circumvented through methodological and site considerations,^42^, ^43^, ^44^ though antecedent hydroclimate extremes (e.g., drought or anomalously wet periods) may mask TC signals in tree rings.

From such paleoclimate perspectives, the storm isotope spike hypothesis^45^ assumes that: (a) TC‐derived rainfall results in significantly lower isotopic compositions during storm landfalls (direct effect) and passages (indirect effect of outer rainfall bands) compared to the annual (noncyclonic) isotope composition for a particular region or site; (b) TC‐derived isotopic compositions are rapidly propagated to surface water (SW, hours to days) and more slowly to GW (months to years), and quickly homogenize in GW reservoirs^46^, ^47^, ^48^; and (c) these isotopic compositions are later incorporated into freshwater and terrestrial (e.g., aquifers, tree rings, pollen, lake sediments, and speleothems) and marine (e.g., corals and ocean sediments) paleoproxies, enabling paleotempestological reconstructions.^41^, ^45^, ^49^, ^50^, ^51^

Nonetheless, reconstructing and interpreting isotope paleoproxies and their further empirical correlation with potential extreme events require us to understand the contemporaneous rainfall isotopic dynamics. Historically, the classical low latitude “amount effect” rationale has been used as a transfer function to evaluate past climate conditions.^34^, ^52^, ^53^, ^54^, ^55^ This isotopic rainfall amount‐dependent relationship is, to a large degree, evaluated considering historical monitoring stations (i.e., the Global Network of Isotopes in Precipitation [GNIP] by the International Atomic Energy Agency [IAEA]),^56^, ^57^ sometimes several hundred kilometers from the actual study area rather than representing local rainfall producing conditions, orographic effects, appropriate interannual variability, and the complete physical understanding of the amount effect mechanism.^31^, ^54^, ^55^, ^58^

While existing long‐term (e.g., over 170 years) and high‐quality climate data have allowed for reasonable climatic characterizations of TC seasons in the North Atlantic and Eastern Pacific Ocean basins, ground‐based rainfall isotopic information for regional or smaller scale characterizations has been lacking. Such information provides insights into the isotopic characteristics of TCs and the propagation of isotopically distinct rainfall pulses across SW bodies and GW reservoirs. The recent development of relatively inexpensive instruments based on laser spectroscopy has improved our ability to achieve a greater temporal and spatial resolution of water tracer data^59^, ^60^; however, such implementation has not yet been widely used in TC‐affected regions such as the North Atlantic and Eastern Pacific Ocean basins.^61^, ^62^ Overall, combining evidence from different archives and proxies allows the development and coverage of more reliable paleoclimate records, which is urgently needed to build scientific evidence based on past spatial TC patterns.

Here, we present the results from an isotope tempestology collaborative research network (2016–present; known as STORM; https://tropicalwaterscr.wixsite.com/hydro/storm) across the North Atlantic and Eastern Pacific Ocean basins. This initiative has been centered mainly on collecting and analyzing recent high‐frequency (hourly to daily) samples of precipitation (P), SW, and GW during TC landfalls and passages to improve the understanding of isotope tempestology processes. For example, the 2020 hurricane season in the North Atlantic was unprecedented, with 30 named TCs, 14 of which impacted the Gulf of Mexico and the Caribbean Sea basins. This study presents storm isotope compositions considering regional (noncyclonic) monitoring efforts with sampling frequencies ranging from daily to monthly between 2013 and 2023. This analysis aims to (a) estimate the contribution of TCs to the seasonal and annual regional precipitation budget, (b) analyze and document the isotopic characteristics of 40 TCs (ranging from tropical depressions/storms to category 5 hurricanes) using recent (2012–2023) and archived (1984–1995) observations, and (c) determine whether the observed isotopic variability from P, SW, and GW during TC events can be used to diagnose hydrological responses and improve paleotempestological reconstructions (i.e., intensity, frequency, relative cyclonic contribution to the regional water cycle). Daily or high‐frequency SW and GW (i.e., streams, rivers, and shallow or deep GW reservoirs) isotope time series for assessing the propagation and incorporation of TC isotopic pulses into freshwater environments are very limited across these regions.^61^, ^62^, ^63^

## DATA AND METHODS

### STORM network and isotopic data

**FIGURE 1:**
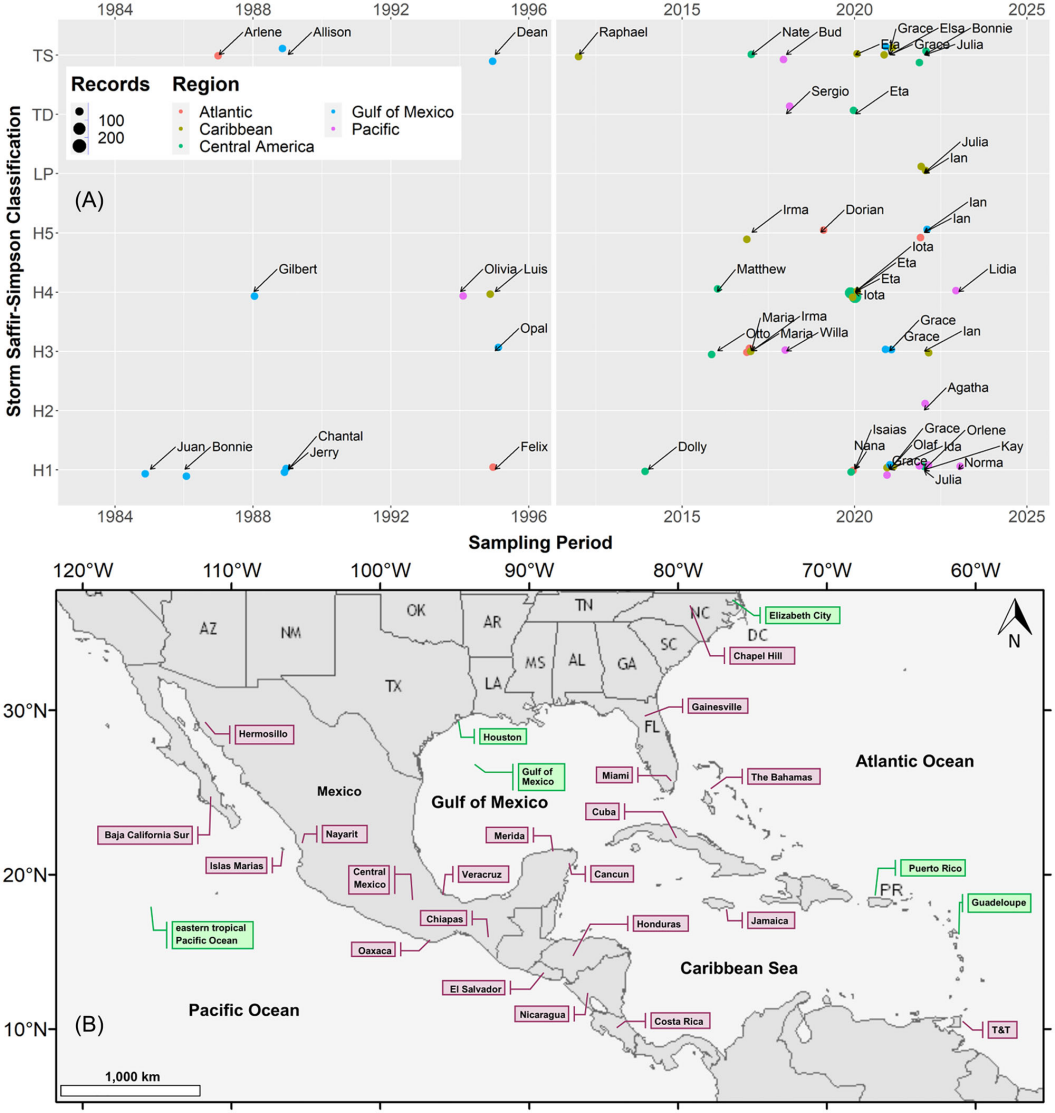
(A) Diagram showing recent (2012–2023) and archived (1984–1995) records of tropical storms (Saffir–Simpson storm classification^103^, ^104^, ^105^) sampled for water isotopes analysis within the North Atlantic, Caribbean Sea, Central America, Gulf of Mexico, and Eastern Pacific Ocean regions. Two storms, Olivia and Opal, included sampling (rainfall and vapor) during aircraft reconnaissance missions.^45^, ^49^, ^64^, ^65^, ^66^ (B) Sampling locations for isotope archives (1985–1995; green labels) and recent (2013–2023) sampling campaigns (purple labels) within the STORM network. Sampling locations in Costa Rica, Honduras, Baja California Sur, Cuba, and Central Mexico included multiple sites.

Recent (2012–2023) and past (1984–1995) storm events were classified by region (i.e., Atlantic, Caribbean Sea, Central America, Gulf of Mexico, and Eastern Pacific), intensity, and sampled for water isotopes analysis (δ^18^O and δ^2^H). Due to the opportunistic nature of this work, difficulties with logistics and access, and short‐term coordination of sampling during TC events (<1 week), the network has relied on citizen‐driven collaboration and existing monitoring stations from nationwide isotope networks, research institutes, national park services, and government and private environmental agencies. A summary of the TC events is shown in Table 1 and Figure 1. To complement this database, other isotope archives (i.e., TCs from 1985 to 1995 and 2012) were included from published and available GNIP records (*N* = 236; Table 1 and Figure 1) as follows: (a) Dean (1995), Luis (1995), and Felix (1995); (b) Olivia (1994; rainfall samples near 750 hPa) and Opal (1995; rainfall and vapor samples near 750 hPa); (c) Jerry (1989), Gilbert (1988), Allison (1989), Chantal (1989), and Arlene (1993); (d) Juan (1985) and Bonnie (1986); and (e) Raphael (2012).^49^, ^50^, ^56^, ^57^, ^64^, ^65^, ^66^ Published and GNIP isotope records were sampled in Guadeloupe Island (Lesser Antilles), Houston coast (Texas, United States), Elizabeth City (North Carolina, United States), Puerto Rico, and air aircraft reconnaissance missions over the Mexican Pacific coast and the Gulf of Mexico. Since archived samples were analyzed mainly via isotope mass ratio spectrometry (IMRS), only 26 archived samples reported both δ^18^O and δ^2^H values.

**TABLE 1:** Summary of historic (1985–1995) and recent (2012–2023) tropical cyclones sampled for water isotope analysis, including the Saffir–Simpson classification,^103^, ^104^, ^105^ storm name, country or sampling domain, year, and the number of available records.

Saffir–Simpson classification	Name	Country/domain	Year	No. records
H1	Juan	US Gulf Coast	1985	2
H1	Bonnie	US Gulf Coast	1986	2
TS	Arlene	US Atlantic Coast	1987	17
H4	Gilbert	US Gulf Coast	1988	23
H1	Chantal	US Gulf Coast	1989	4
H1	Jerry	US Gulf Coast	1989	2
TS	Allison	US Gulf Coast	1989	12
H4	Olivia	Mexico	1994	26
H1	Felix	US Atlantic Coast	1995	10
H3	Opal	US Gulf Coast	1995	68
H4	Luis	Puerto Rico	1995	22
TS	Dean	US Gulf Coast	1995	46
TS	Raphael	Guadalupe	2012	2
H1	Dolly	Costa Rica	2014	1
H3	Otto	Costa Rica	2016	59
H4	Matthew	Costa Rica	2016	10
H3	Irma	Bahamas	2017	7
H3	Maria	Cuba	2017	2
H3	Maria	Bahamas	2017	18
H5	Irma	Cuba	2017	19
TS	Nate	Costa Rica	2017	27
H3	Willa	Mexico	2018	1
TD	Sergio	Mexico	2018	7
TS	Bud	Mexico	2018	1
H5	Dorian	Bahamas	2019	26
H1	Isaias	Bahamas	2020	15
H1	Nana	Honduras	2020	4
H4	Eta	Honduras	2020	135
H4	Eta	Nicaragua	2020	55
H4	Eta	Costa Rica	2020	13
H4	Eta	Costa Rica	2020	4
H4	Iota	Honduras	2020	96
H4	Iota	Nicaragua	2020	12
H4	Iota	Costa Rica	2020	14
H4	Iota	Costa Rica	2020	10
TD	Eta	El Salvador	2020	5
TS	Eta	Jamaica	2020	4
H1	Grace	Mexico	2021	7
H1	Grace	Mexico	2021	1
H1	Ida	Cuba	2021	12
H1	Olaf	Mexico	2021	1
H3	Grace	Mexico	2021	15
H3	Grace	Mexico	2021	1
TS	Elsa	Cuba	2021	25
TS	Grace	Jamaica	2021	13
TS	Grace	Mexico	2021	4
H1	Julia	Honduras	2022	29
H1	Julia	Costa Rica	2022	18
H1	Kay	Mexico	2022	5
H1	Orlene	Mexico	2022	2
H2	Agatha	Mexico	2022	26
H3	Ian	Cuba	2022	16
H5	Ian	US Atlantic Coast	2022	2
H5	Ian	US Gulf Coast	2022	3
LP	Ian	Trinidad and Tobago	2022	2
LP	Julia	Trinidad and Tobago	2022	2
TS	Bonnie	Honduras	2022	10
TS	Bonnie	Costa Rica	2022	25
TS	Julia	El Salvador	2022	7
H1	Norma	Mexico	2023	4
H4	Lidia	Mexico	2023	3

Abbreviations: H1, H2, H3, H4, and H5, hurricanes categories; LP, low pressure; TD, tropical depression; TS, tropical storm.

Recent samples (*N* = 778) correspond to concerted STORM monitoring efforts of 27 storms (Table 1 and Figure 1). These sampling efforts involved monitoring sites in Trinidad and Tobago (Diego Martin), Cuba (La Habana and Cienfuegos), Jamaica (Kingston), The Bahamas (Nassau), Central America (Costa Rica, Nicaragua, El Salvador, and Honduras), Mexico (Pacific, Gulf of Mexico, and Caribbean Sea coasts), Florida (Miami and Gainesville), and North Carolina (Chapel Hill). The best available TC trajectory tracks from the North Atlantic (1851–2023), the Northeast, and the North Central Pacific (1949–2023) were obtained from the second version of the HURricane DATabase (HURDAT2)^67^, ^68^ (see Figures S1–S3). Overall, TC samples correspond to the low‐pressure system (LP, 0.4%), tropical depression (TD, 1.2%), tropical storm (TS, 19.0%), and hurricane categories H1 (11.7%), H2 (2.6%), H3 (18.4%), H4 (41.7%), and H5 (4.9%; Table S1).

Regional long‐term monitoring (noncyclonic) of daily and monthly rainfall samples (*N* = 7229; 2013–2023) include sites in Trinidad and Tobago (Gran Couva), Cuba (La Habana and Cienfuegos), Central America (Costa Rica, Nicaragua, El Salvador, and Honduras), and Mexico (Pacific, Caribbean, and Gulf of Mexico coasts; Table 1 and Figure 1). Complementary regional data for the Dominican Republic (Santo Domingo) and Guatemala (Platanar) were obtained from GNIP (GNIP, 2024; Table S2). Tables S1 and S2 are also available at the HydroShare^69^ online repository.

### Sample collection and isotopic analysis

#### Precipitation

Event and long‐term monitoring samples were collected in passive devices (Palmex RS1, RS2, and in‐house collectors).^70^ Samples were transferred and stored in airtight high‐density polyethylene (HDPE) and borosilicate containers at 5°C until laser spectroscopy analysis in multiple laboratories (analyzer specification in parentheses): Costa Rica (L‐2120‐*i* and LWIA‐45‐EP), Nicaragua (LWIA‐45‐EP), Honduras (L‐2130‐*i*), El Salvador (L‐2130‐*i*), México (L‐2120‐*i* and LWIA‐45‐EP), Cuba (IRMS), Trinidad and Tobago (TWIA‐45‐EP), United States (TWIA‐GLA431). Stable isotope abundances are expressed as δ^18^O or δ^2^H = (R_s_/R_std_ − 1) × 1000, where R is the ^18^O/^16^O or ^2^H/^1^H ratio in a sample (s) or standard (std) and reported in the delta‐notation (‰–per mil) relative to Vienna Standard Mean Ocean Water (V‐SMOW) reference standards. Deuterium excess (hereafter, *d*‐excess) was calculated as *d*‐excess = δ^2^H − 8·δ^18^O.^52^

#### Surface water and groundwater

SW and GW samples (springs and cave infiltration; *N* = 2599) were collected manually and using automated samplers in Costa Rica and Honduras. Samples in north‐central Costa Rica, near Cerro Dantas (Flores Spring and Quebrada Grande Stream) and Sacramento (Sacramento Spring), were collected on a subdaily, daily, and weekly basis using Sigma 900 MAX and AS950 autosamplers (HACH, United States). Samples in north Pacific Costa Rica, near Liberia station (Tempisque River), were collected weekly and daily during the events. Cave drip water at Venado Caves (northern Costa Rica) was collected every week using an in‐house passive collector (Palmex‐type device). In Honduras, samples were collected manually on weekly, daily, and subdaily (every 2 h) bases at Catacamas (Talgua River) and subdaily basis at El Cajón Dam (Table S3). All SW and GW samples were poured without head space into 30 mL HDPE bottles and were stored at 5°C, hermetically sealed with Parafilm, until isotopic analysis. Table S3 is also available at the HydroShare^69^ online repository.

The damping ratio (*D*) of the rainfall isotope composition in the observed SW and GW isotopic response was calculated as the ratio of the standard deviation of stream/river water or spring isotopic composition (i.e., all data points; SD_s_) to the standard deviation of precipitation isotopic composition (SD_p_; *D* = SD_s_/SD_p_) [−, unitless].^71^, ^72^, ^73^ This versatile parameter provides information about the degree of water mixing in the watershed and strongly correlates with the mean transit time.^74^, ^75^ In general, lower *D* ratios denote longer mean transit times and large mixing with GW sources, whereas large *D* ratios represent quicker catchment responses and less mixing with GW systems. In addition, the daily isotopic ratio between precipitation and SW/GW—P/SW and P/GW [−, unitless]—was calculated to evaluate the influence of TC isotopic excursions.^46^ This ratio allows for the determination of annual isotope cycles in streams, rivers, springs, and cave systems, as well as the identification of isotope “spikes” (e.g., larger ratios) due to TC‐derived rainfall. Cumulative distribution function (CDF) curves were calculated based on P/SW and P/GW [−] ratios.

### Regional climatic analysis

#### Contributions of tropical cyclones to regional precipitation

We estimated the contribution of TCs to local/regional precipitation using daily precipitation data from the Climate Hazards Group Infrared Precipitation with Station Data (CHIRPSv2)^76^ and the Multi‐Source Weighted‐Ensemble Precipitation version 2 (MSWEPv2)^77^ between 1984 and 2023. CHIRPS and MSWEP integrate satellite imagery data with in situ weather stations at 0.05° (∼6 km) and 0.1° (∼11 km), respectively. We selected these precipitation products because they provide daily precipitation data at high spatial resolution, are updated regularly, and perform reasonably well in Central America and the Caribbean Islands compared to other high‐resolution gridded precipitation products in the Caribbean.^78^ In the Americas, the hurricane season spans from May 15th to November 30th (https://www.aoml.noaa.gov/hrd‐faq/). We divided the analysis for the 1984–1995 and 2012–2023 periods to be consistent with the isotopic data (see the section “STORM network and isotopic data”) and analyzed the 1984–2023 period to have a better climatic representation. While it is often preferred to use weather station data instead of remote‐sensed rainfall estimates,^79^ the lack of long‐term and high‐quality data prevented us from relying solely on weather stations for this analysis.

In this work, we included only tropical storms and hurricanes as TCs. The rationale for not including tropical depressions (i.e., unnamed TCs) is that they are unlikely to have a broad rainfall field based on TC‐associated circulation.^79^ This work did not include subtropical and extratropical storms. To determine the tracks of the selected TCs, we used the HURDAT2 dataset for the North Atlantic and Eastern Pacific Ocean basins. The selected TCs were those whose center crossed at most 500 km from land. This radial distance has been shown to be appropriate for analyzing TC contributions to local precipitation^79^, ^84^ since it is the average radius of TC‐associated circulation and rainbands. Given that our estimates only included precipitation over 500 km from the center of the TC as TC‐associated precipitation, this may underestimate (overestimate) TC precipitation from TCs with a radius greater (smaller) than 500 km. This radius may also include precipitation from non‐TC synoptic systems such as fronts, but mostly over the United States. However, we acknowledge that TC‐precipitation radius evolves along with the storm (e.g., with changes in its intensity), affecting the TC rainfall field.^80^ Also, since the HURDAT2 data have a 6‐h frequency, we selected the 6‐h mean location of the center of the storms each day. Finally, we assessed each TC's contribution to rainfall as the fraction of a TC's‐derived precipitation over the total precipitation from monthly, seasonal, and annual contributions.

### Statistical analysis

The median values of δ^18^O and *d*‐excess per domain (i.e., atmospheric, coastal, inland, and maritime) were compared against the regional median values as a reference group (Wilcoxon test) using the R package *rstatix*.^81^ Similarly, pairwise δ^18^O and *d*‐excess comparisons between storm categories were computed using the R package *rstatix*.^81^ Isotope data visualizations were computed using the R packages *ggplot2* and *ggbreak*.^82^, ^83^

## RESULTS

### TC's precipitation contribution to the seasonal and annual regional water budget

**FIGURE 2:**
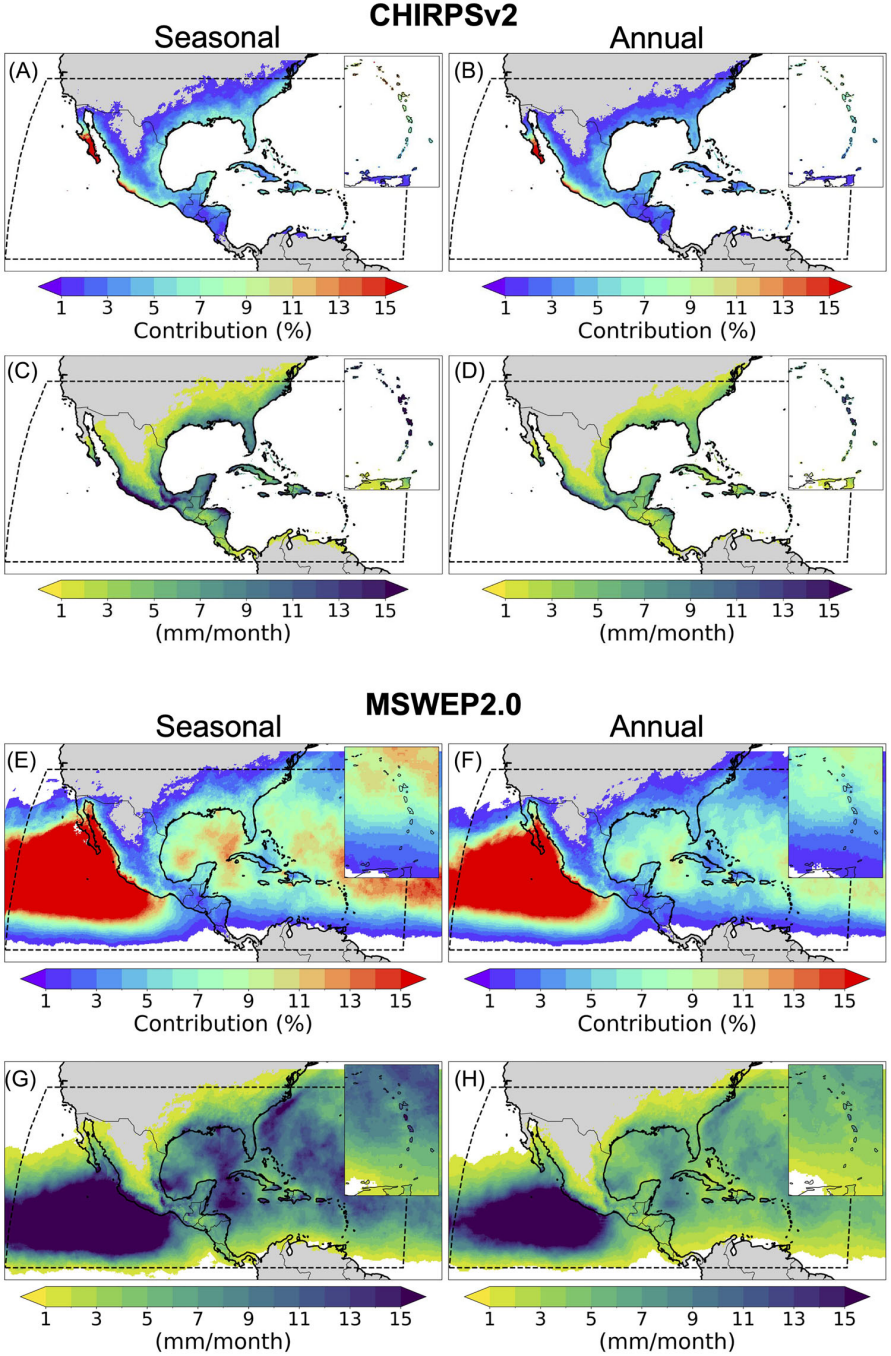
Tropical cyclone contributions to precipitation in the study region. Panels A/B and E/F show the seasonal and annual mean contributions (as percentages) to rainfall (1984–2023) from Climate Hazards Group Infrared Precipitation with Station Data (CHIRPS) and Multi‐Source Weighted‐Ensemble Precipitation (MSWEP), respectively. Both products are consistent with relative contributions. The area with the highest contributions (>15%) is Baja California Peninsula and portions of the western coast of Mexico. Panels C/D and G/H show the seasonal and annual mean precipitation from tropical cyclones (in mm/month) from both datasets. While a similar pattern with the relative contributions is observed, the actual rainfall amounts are lower in Baja California, highlighting the importance of tropical cyclones as a source of moisture for this area. The areas with contributions below 1% were masked for visual purposes.

Figure 2 shows TC contributions to seasonal and annual mean precipitation across the study area between 1984 and 2023, as estimated using CHIRPS and MSWEP. In CHIRPS, seasonal mean contributions varied from less than 1% to 54%, with 4% on average. The highest relative contributions were in Baja California, with over 20%, and the western coast of Mexico, with ∼16%, on average (Figure 2A,B). The lowest contributions were in north‐central Mexico, northern South America, and the US continental interior, with 1%–2% of the average seasonal and annual rainfall coming from TCs (Figure 2A,B). Estimates from MSWEP are similar to CHIRPS in magnitude and spatial patterns (Figure 2E,F), but TC contributions are slightly over Baja California (e.g., ∼60%). These results are consistent with previous observational and modeling studies in the study area in terms of the magnitude of TC contributions to precipitation and spatial patterns.^28^, ^79^, ^80^, ^84^ Whereas the greatest contributions were in northwestern–western Mexico, the highest TC‐associated rainfall rates in seasonal and annual means were on the southern coast of Mexico in CHIRPS and MSWEP (Figure 2C,D and G,H). The highest average annual precipitation rate was over 17 mm/month, whereas the highest rainfall amounts on seasonal means were ∼30 mm/month (Figure 2D,H). These data indicate that TCs are an important source of moisture for northwestern Mexico and southwestern United States. However, while CHIRPS and MSWEP agree in these patterns, seasonal and annual estimates using MSWEP are slightly lower over the isthmus of Tehuantepec (Mexico; Figure 2C,D and G,H).

**FIGURE 3:**
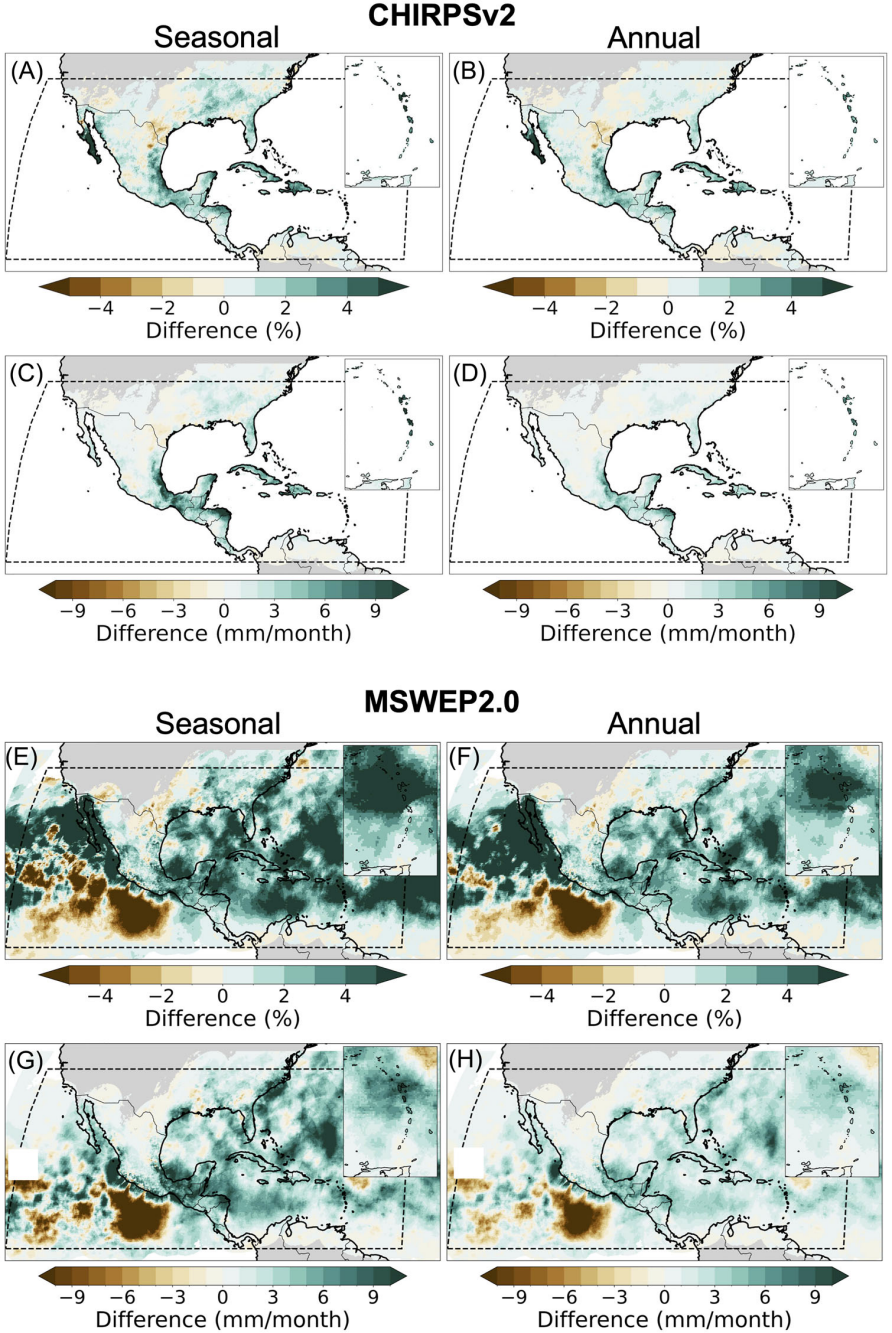
Changes in tropical cyclone contributions to precipitation. Panels A and B show the differences in tropical cyclone contributions between the periods 1984–1995 and 2012–2023. In most of the study area, the contributions for the 2012–2023 period are higher compared to 1984–1995, except in southern Central Plains, where the opposite occurred. Panels C and D are similar to the A and B panels but for changes in tropical cyclone rainfall amounts. The areas with contributions below 1% were masked for visual purposes. CHIRPSv2, Climate Hazards Group Infrared Precipitation with Station Data; MSWEP2.0, Multi‐Source Weighted‐Ensemble Precipitation version 2.

By comparing the TC contributions to seasonal and annual rainfall between the 1984–1995 and 2012–2023 periods from CHIRPSv2 and MSWEP2, there was a noticeable increase in TC‐associated rainfall in the later period (Figure 3). For example, as shown in Figure 3A,E, seasonal contributions for the 2012–2023 period were up to 30% greater than the 1984–2012 period in the Baja California Peninsula in CHIRPSv2 and 39% in MSWEP2. Similarly, annual contributions were 24% greater in the 2012–2023 period in CHIRPSv2 and 29% in MSWEP2 (Figure 3B,F). It is noteworthy that the 2012–2023 period included the most active hurricane season in the North Atlantic (2020), which could explain these results. However, a decrease in TC‐associated rainfall occurs in the US southern Central Plains, decreasing −6% and −4% on seasonal and annual means, respectively, in CHIRPSv2 (Figure 3A,B). A similar result is observed for MSWEP2 over land. However, −37% and −22% occur over the eastern Pacific on seasonal and annual means, respectively (Figure 3E,F). These results are consistent with changes in TC‐associated rainfall rates (Figure 3C,D and G,H), with an increase of up to 26 mm/month in 2012–2023 in CHIRPSv2 and 29 mm/month in MSWEP2. However, in contrast to the relative contributions, the greatest increase in seasonal TC‐associated precipitation occurred in Central America and southern Mexico (Figure 3C,G). A similar but slightly lower spatial pattern is observed in TC annual mean precipitation (15 mm/month). As with the relative contributions, a decrease in TC rainfall occurred in the US southern Central Plains (Figure 3C,D and G,H).

### Regional (noncyclonic) and tropical cyclones isotope variability

**FIGURE 4:**
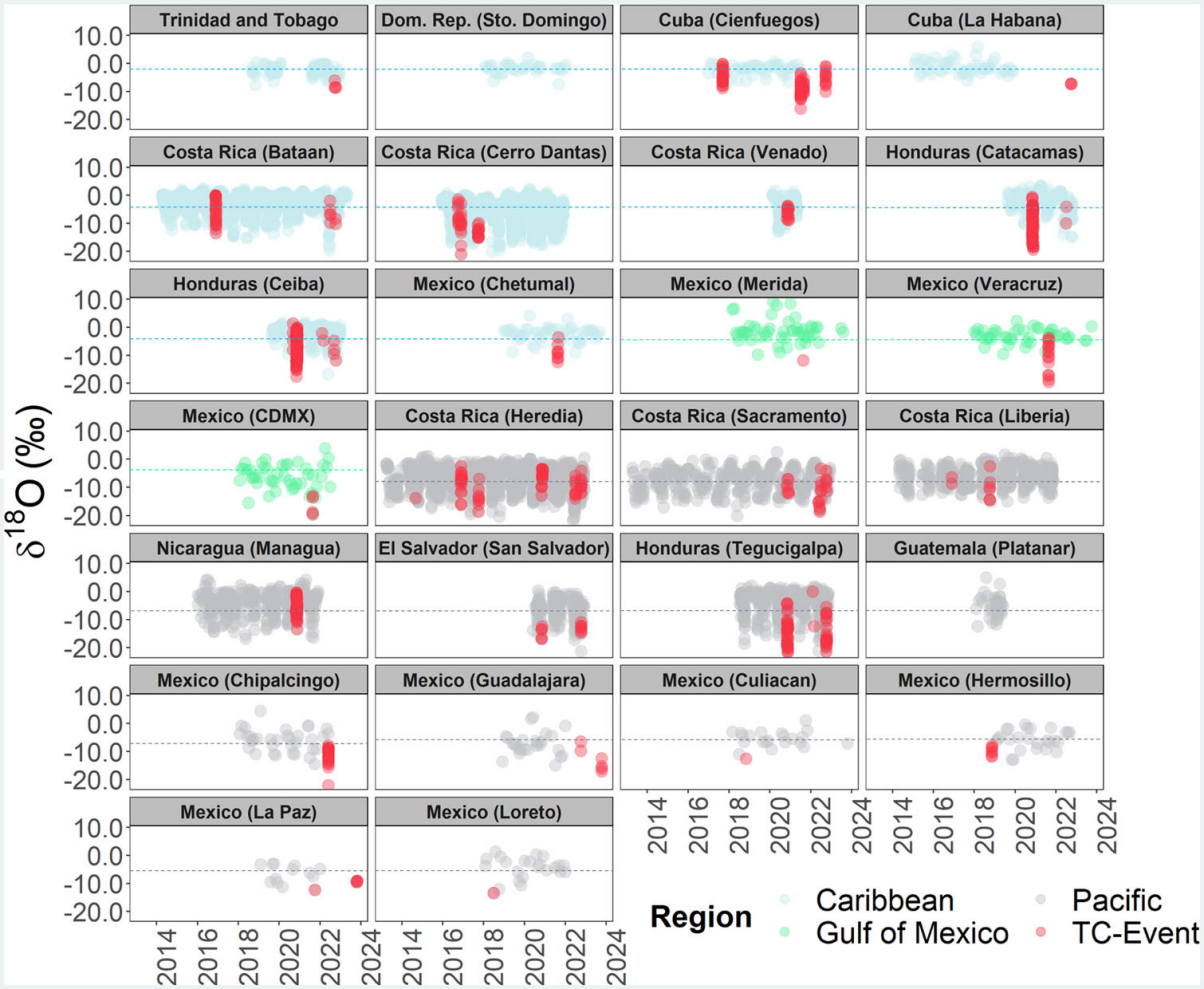
Regional long‐term δ^18^O (‰) rainfall compositions (*N* = 7229) across the Gulf of Mexico, Caribbean Sea, Central America, and Mexico (color‐coded) between 2013 and 2023. Red dots (*N* = 778) correspond to recent tropical storm event samples (tropical cyclone [TC]‐event). Long‐term isotope monitoring denotes daily, weekly, and monthly sampling. Complementary samples (Guatemala, Platanar and the Dominican Republic, Santo Domingo) were obtained from Global Network of Isotopes in Precipitation (GNIP). Color‐coded dashed lines denote the long‐term mean per domain, as described in “Regional (noncyclonic) and tropical cyclones isotope variability” section.

Figure 4 compares rainfall δ^18^O (‰) compositions between regional (long‐term monitoring sites and noncyclonic) and TCs across the Gulf of Mexico, Caribbean Sea, and Eastern Pacific (2013–2023). Annual δ^18^O (‰) compositions in the Caribbean Sea were commonly characterized by near uniform high δ^18^O values throughout the hydrological year, ranging from +5.92 (‰) to −7.85 (‰), with a mean value of −2.14 (±2.02‰). Although TCs across the Caribbean Sea can produce low δ^18^O values (e.g., −16.2‰, Elsa, 2021), δ^18^O compositions were, in general, right skewed toward high values, with a mean value of −7.13 (±3.30‰). In the Gulf of Mexico region, a similar pattern was observed with a greater isotope variability during the wet season (May–November), ranging from +9.01 (‰) to −19.32 (‰), with a mean value of −3.57 (±4.35‰). TCs across this region can also generate low δ^18^O values, up to −19.57 (‰; e.g., Grace, 2022), with a mean value of −11.67 (±5.30‰).

In the Caribbean slope of Central America and Mexico, annual δ^18^O compositions exhibited a typical W‐shaped pattern, with low values during the wet season (May–November; up to ∼−15‰), with a mean value of −4.10 (±3.88‰). TCs across this region can result in very low δ^18^O values, up to −21.1 (‰; e.g., Otto, 2016), with a mean value of −8.21 (±4.61‰). In the central and Pacific intramountainous regions of Central America and Mexico, the rainy season was also characterized by a marked W‐shaped isotope variability with lower δ^18^O compositions than the Caribbean slope during the wet season, typically up to ∼−17 (‰). TC passages across this region can result in low δ^18^O values up to −21.73 (‰; e.g., Iota, 2020). In the central and northern Pacific coast of Mexico, annual δ^18^O compositions were characterized by more uniform values, ranging from −14.96 (‰) to +4.47 (‰) with a mean value of −5.92 (±3.62‰). In general, TCs over this region resulted in lower δ^18^O values (e.g., −22.1‰; Agatha, 2022) compared to the annual compositions.

**FIGURE 5:**
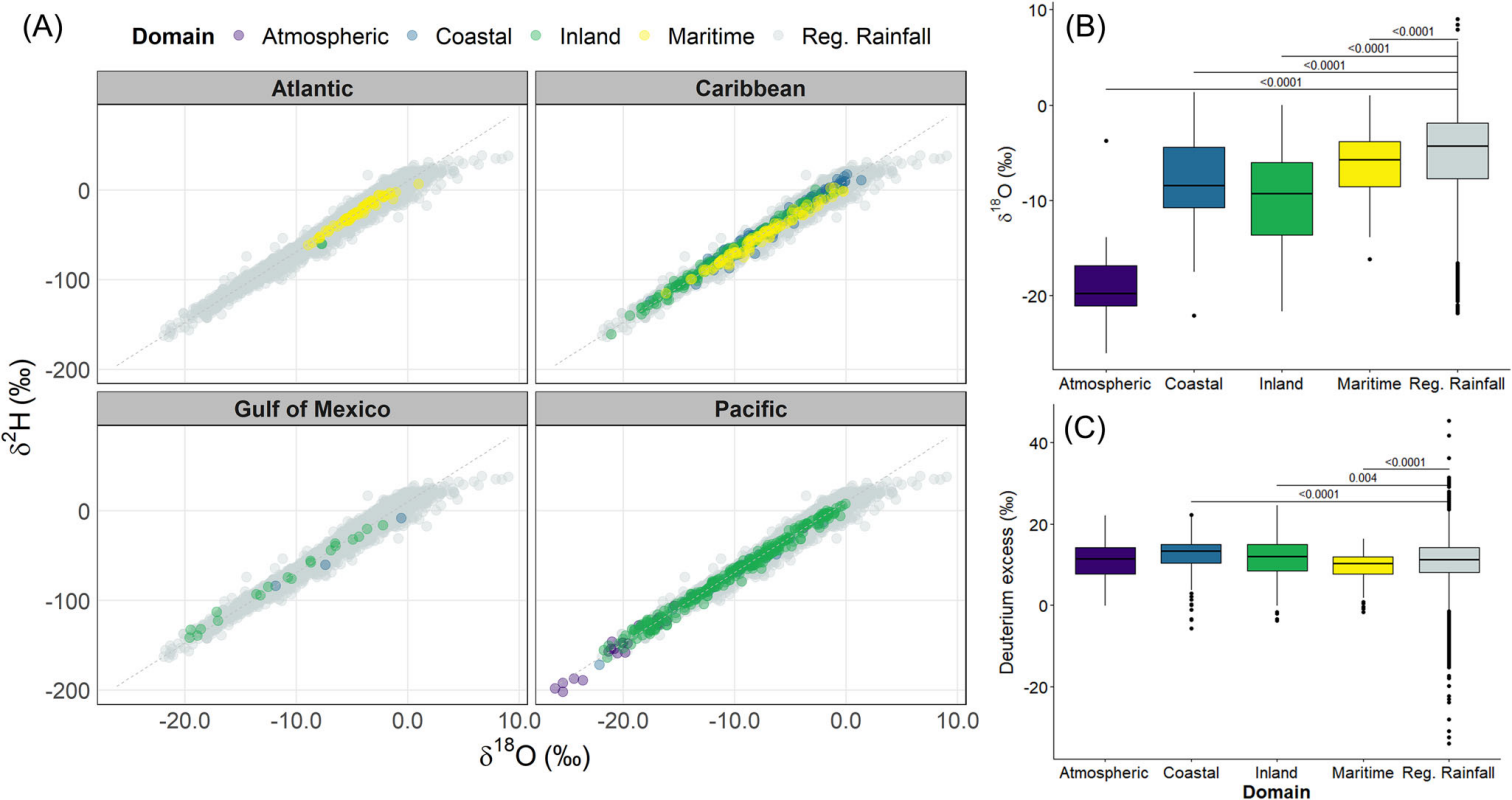
(A) Dual isotope plots color‐coded and grouped per main region and different sampling domains. The gray dashed line denotes the regional meteoric water line (δ^2^H = 7.88⋅δ^18^O + 10.22; Adj. *R* ^2^ = 0.98; *N* = 7229; 2013–2023). (B, C) Box plots comparing tropical cyclones (TCs) δ^18^O (‰) and *d*‐excess (‰) variability per sampling domain with the regional‐wide rainfall isotope compositions (2013–2023) as reference group. Brackets show only significant *p* values based on the Wilcoxon test. The atmospheric domain includes rain and vapor samples collected between 480 hPa and 700 hPa during aircraft reconnaissance missions.

Figure 5A shows dual isotope plots for the main geographical regions (North Atlantic, Caribbean, Gulf of Mexico, and Eastern Pacific) and sampling domains (atmospheric, coastal, inland, and maritime). In general, coastal, inland, and maritime sampling domains plotted closely along the regional meteoric water line (δ^2^H = 7.88⋅δ^18^O + 10.22; Adj. *R* ^2^ = 0.98; *N* = 7229; 2013–2023). Rainfall samples (e.g., Olivia, 1994 at 3 km above the surface) showed low values coinciding with the lower limit of the inland domain in the Eastern Pacific region. Opal (1995) archives only included δ^18^O values, limiting the dual space analysis and *d*‐excess estimations. Coastal and maritime isotope compositions exhibited high values, whereas inland interactions (e.g., mountain ranges) produced low values within the range of the regional isotope dual space. Overall, δ^18^O median values from all sampling domains were significantly different (*p* < 0.0001) from the regional (noncyclonic) δ^18^O median value and revealed consistently low compositions (Figure 5B). In terms of *d*‐excess, all domains were significantly different (*p* < 0.0001–0.004) from the regional *d*‐excess median value (+11.1‰; Figure 5C). Maritime *d*‐excess values were closer to the global equilibrium value (+10‰), while coastal and inland *d*‐excess were higher. Water vapor *d*‐excess values were not significantly different from the regional pattern, but these measurements were limited to only one TC (Olivia, 1994).

### Tropical cyclones δ^18^O and *d*‐excess variability

**FIGURE 6:**
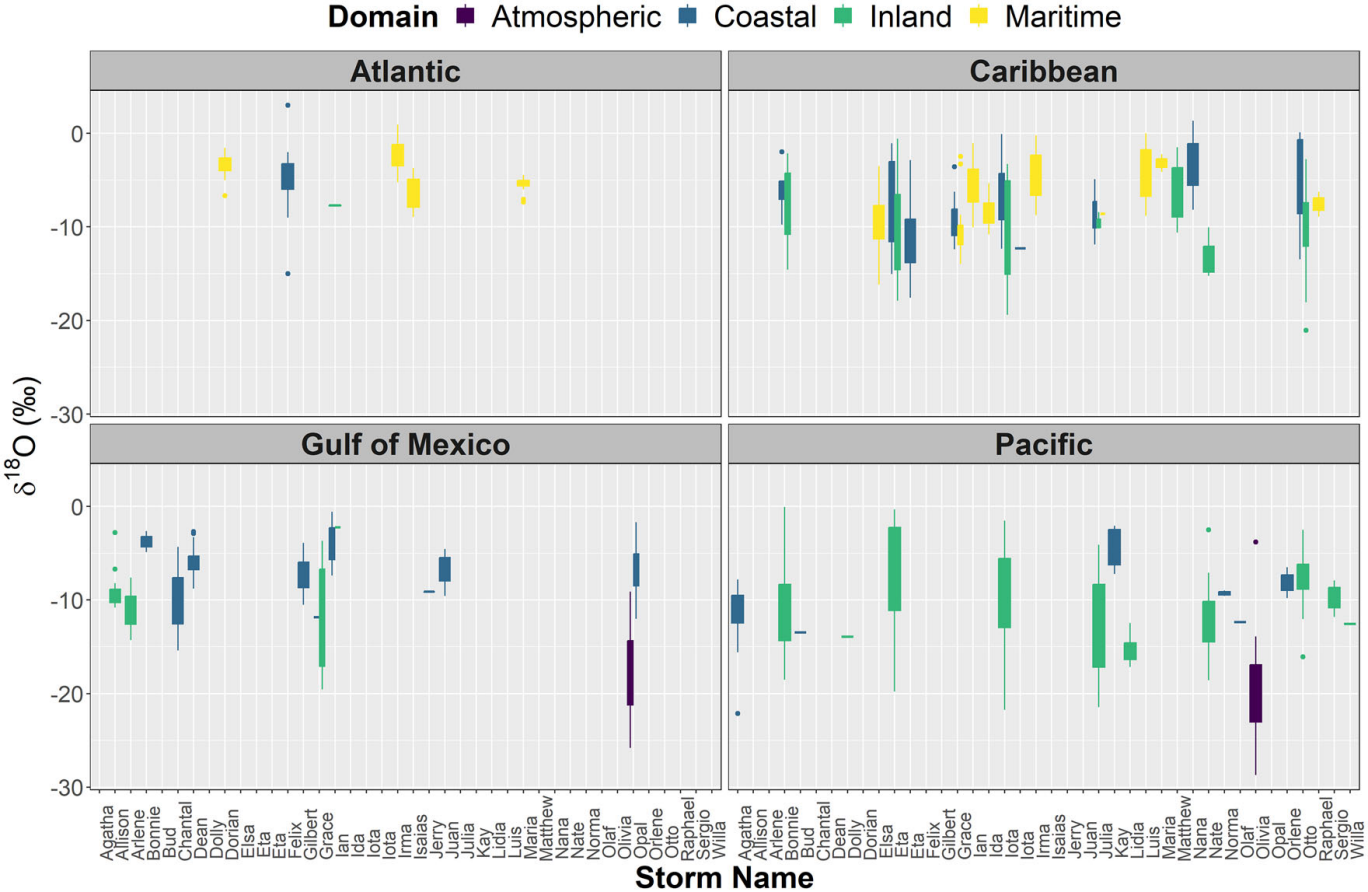
Boxplots showing δ^18^O (‰) distribution for tropical storms (see Figure 1) across the North Atlantic, Gulf of Mexico, Caribbean Sea, and Eastern Pacific Ocean basins. Color‐coded domains correspond to maritime (islands), inland (continental), coastal (continental), and atmospheric (rainfall and water vapor during aircraft reconnaissance missions).

**FIGURE 7:**
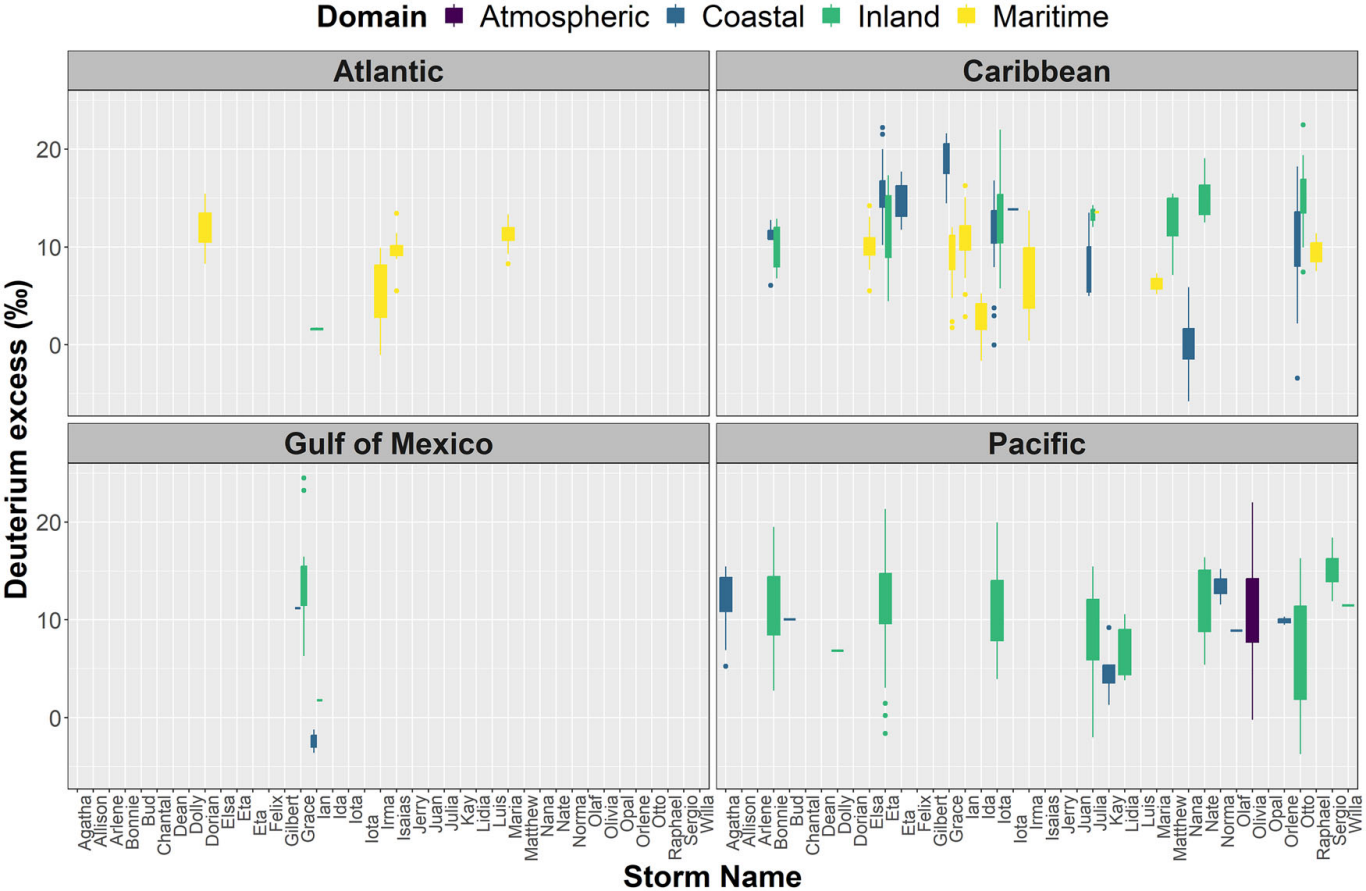
Boxplots showing deuterium excess (‰) distribution for tropical storms (Figure 1) across the North Atlantic, Gulf of Mexico, Caribbean Sea, and Eastern Pacific Ocean basins. Color‐coded domain corresponds to maritime (islands), inland (continental), coastal (continental), and atmospheric (rainfall and water vapor during aircraft reconnaissance missions). Several archived records only contain δ^18^O (‰), limiting *d*‐excess calculations mainly in the Gulf of Mexico domain.

Figures 6 and 7 show δ^18^O and *d*‐excess variability per storm across the North Atlantic, Caribbean Sea, Gulf of Mexico, and Eastern Pacific regions. Overall, TCs across the North Atlantic region were characterized by high δ^18^O values, ranging from +3.00 (‰) to −9.00 (‰), with a mean value of −4.55 (±2.17‰). Only one sample during Felix (1995) exhibited a low value (−15.0‰) in the coastal domain of North Carolina (United States). Mean *d*‐excess in the North Atlantic region was close (+10.34 ± 3.24‰) to the global mean, ranging from −1.03 (‰) to +15.45 (‰).

Similar to the North Atlantic region, δ^18^O compositions in the Caribbean Sea were characterized by a higher mean value (−6.97 ± 3.55‰). Coastal and inland domains exhibited low δ^18^O values, −7.11 (±4.22‰) and −10.11 (±4.70‰), respectively. Land and TC interaction during Otto (2016), Eta (2020), and Iota (2020) resulted in low δ^18^O values, −21.06 (‰), −17.28 (‰), and −19.39 (‰), respectively. In the Caribbean region, maritime *d*‐excess ranged from −1.63 (‰) to +16.25 (‰), with a mean value of +8.31 (±4.02‰). Coastal (−5.77‰ to +22.20‰) and inland (+4.44‰ to +22.49‰) domains presented similar greater mean values, +12.70 (±4.65‰) and +12.91 (±3.65‰), respectively.

In the Gulf of Mexico, Opal (1995) exhibited a mean vapor and rainfall δ^18^O value of −17.40 (±4.11‰), ranging from −9.10 (‰) to −25.80 (‰). Coastal (−0.58‰ to −15.40‰) and inland (−2.23‰ to −19.57‰) δ^18^O compositions showed a similar Caribbean pattern, with mean values of −6.62 (±2.33‰) and −10.61 (±4.06‰). Grace (2021) resulted in low compositions across Mexico's inland. Only two TCs over the Gulf of Mexico (Grace, 2021 and Ian, 2022) reported δ^18^O and δ^2^H values to evaluate *d*‐excess, ranging from −3.59‰ to +24.5‰ with a mean value of +11.45 (±6.58‰).

In the Pacific region, Olivia (1994) exhibited a mean vapor δ^18^O value of −19.81 (±4.94‰), ranging from −3.80‰ to −28.70‰. Olivia's mean *d*‐excess (+10.80±5.79‰) was close to the global mean, varying from −0.20 (‰) to +22.00 (‰). Coastal (−2.07‰ to −22.12‰) and inland (−0.06‰ to −21.73‰) δ^18^O compositions showed lower values compared to the Caribbean and North Atlantic regions, with mean values of −10.26 (±3.50‰) and −9.55 (±5.38‰). Eta (2020; −19.78‰), Iota (2020; −21.73‰), Agatha (2022; −22.12‰), Julia (2022; −21.43‰), and Bonnie (2022; −18.52‰) resulted in the lowest compositions in the Pacific region. Coastal (+1.30‰ to +15.46‰) and inland (−3.73‰ to +21.33‰) domains presented near equilibrium *d*‐excess values, +11.29 (±3.50‰) and +10.82 (±4.67‰), respectively.

### Tropical storm category and isotope variability

**FIGURE 8:**
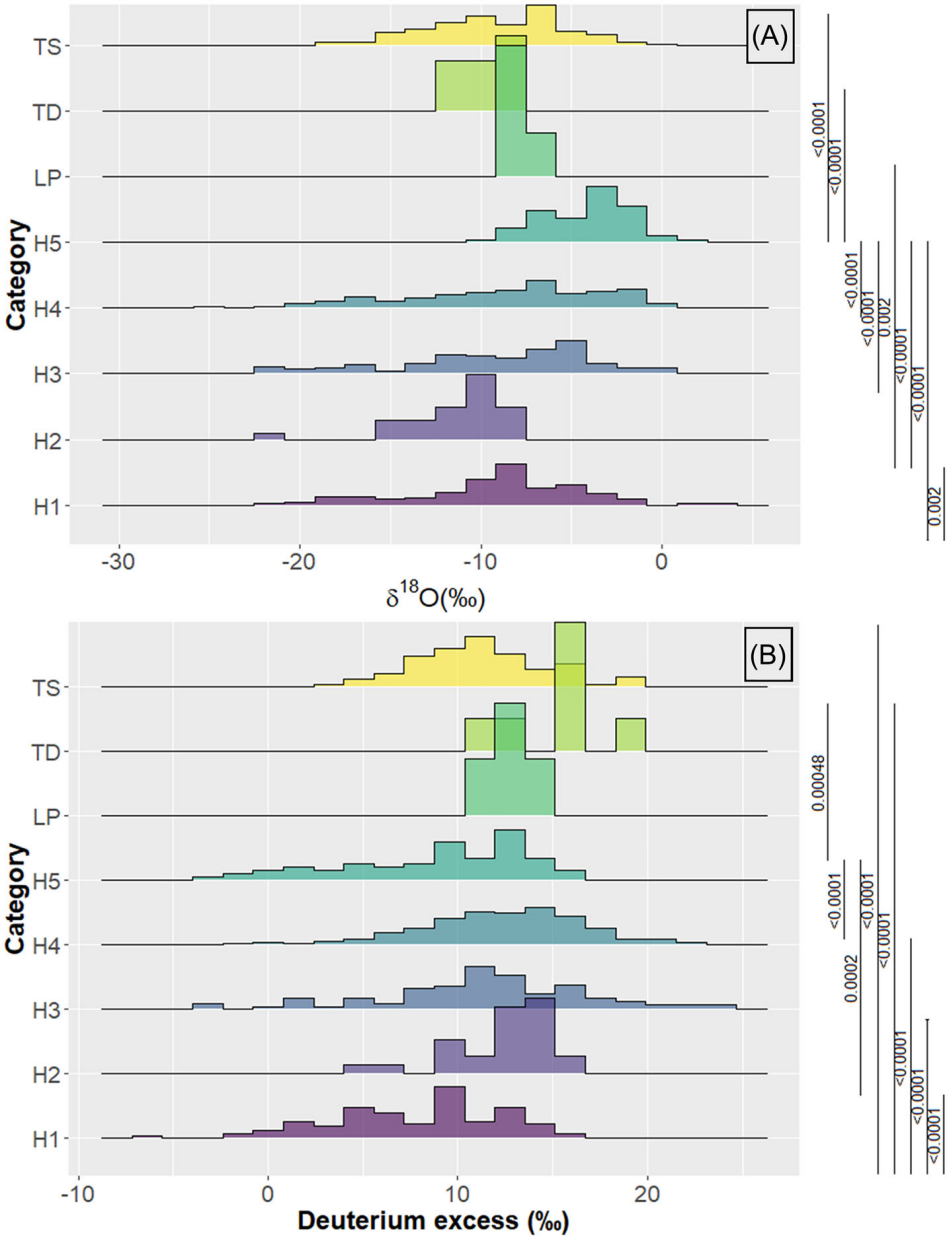
Density distribution plots showing (A) δ^18^O (‰) and (B) *d*‐excess (‰) variability per storm category. Marginal brackets show only significant *p* values based on the Wilcoxon test (pairwise comparison across all storm categories). Hurricanes category: H1, H2, H3, H4, and H5. Tropical depression, storm, and low‐pressure systems: TD, TS, and LP.

Figure 8 shows δ^18^O and *d*‐excess variability from low‐pressure systems (LP) to hurricanes category 1–5 (H1 to H5). No significant δ^18^O differences were found between LP, TD, and TS events. TS exhibited greater δ^18^O variability than LP and TD (Figure 8A). TD and TS were represented by lower median δ^18^O compositions than LP. TD and TS median δ^18^O values were significantly different from H5 (*p* < 0.0001), whereas LP was significantly different from H2 events (*p* = 0.002). Among all hurricane categories, H2 and H5 events were denoted by low and high δ^18^O compositions, respectively. However, H5 events were significantly different (*p* < 0.0001) from the H1, H2, H3, and H4 categories. H1, H3, and H4 categories exhibited similar median δ^18^O compositions with no significant difference, with lower values recorded during H3 and H4 events (Figure 8A).

The lowest *d*‐excess (<0‰) values were recorded during H1, H3, H4, and H5 events (Figure 8B). Category H1 and H5 showed the lowest median *d*‐excess values (<+10‰), whereas in H2–H4 events, median *d*‐excess was consistently above +10 (‰). LP and TD events showed greater median *d*‐excess values than all hurricane categories, except for H2. H1 median *d*‐excess was significantly different from H2, H3, H4, TD, and TS (*p* < 0.0001). H5 median *d*‐excess was significantly different from H2, H4, and TD events (*p* < 0.0001–0.00048). Large *d*‐excess values (up to +20‰) were recorded in H3, H4, and TS events.

### Surface water and groundwater variability during landfall and passage of tropical cyclones

**FIGURE 9:**
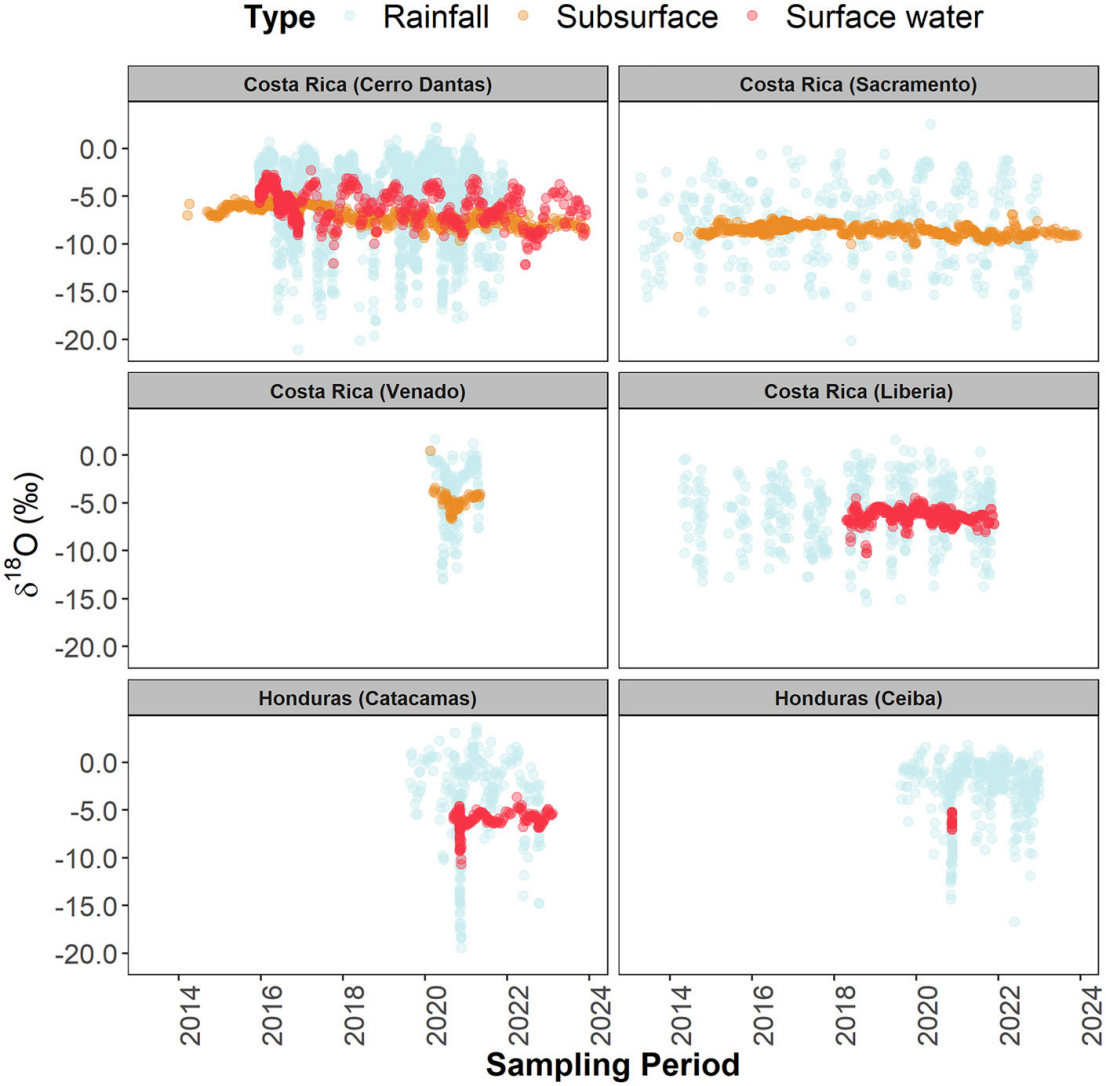
Precipitation (light blue), surface water (red), and subsurface (orange) water δ^18^O (‰) time series (*N* = 6013) across Costa Rica (*N* = 5812) and Honduras (*N* = 201) between 2013 and 2023. Light blue dots correspond to long‐term rainfall isotope monitoring.

Figure 9 shows SW and GW isotope variability across four streams/rivers and three spring/cave systems in north/central Costa Rica and southeastern Honduras. Overall, the rainfall isotope composition exhibited a bimodal pattern (W‐shaped type) throughout the year. Isotopic compositions in SW and GW (to a lesser degree) clearly depicted the isotopic variations in the rainfall modes under distinct damping (D) ratios. The lower *D* ratios indicate longer mean transit times and larger mixing with GW sources, whereas the higher *D* ratios denote faster catchment responses and less mixing with GW.

In Costa Rica, high‐elevation spring systems (>2000 m a.s.l.) exhibited the lowest *D* ratios, ranging from 0.12 (Sacramento Spring) to 0.24 (Flores Spring). The Venado Cave karst system, located in the northern region of Costa Rica, resulted in a larger *D* ratio (0.31) compared to the springs. Quebrada Grande, a dynamic mountainous first‐order stream, exhibited the greatest *D* ratio (0.43), whereas the Tempisque River (a large basin) resulted in a smaller *D* ratio (0.25). In Honduras, the lowest *D* ratio (0.18) was obtained from the El Cajón Dam release during the direct impact of Eta (2020) and Iota (2020). The Talgua River, in the southeastern region of Honduras (characterized by a high‐elevation karst aquifer system and agricultural lowlands), resulted in a slightly greater *D* ratio (0.21).

**FIGURE 10:**
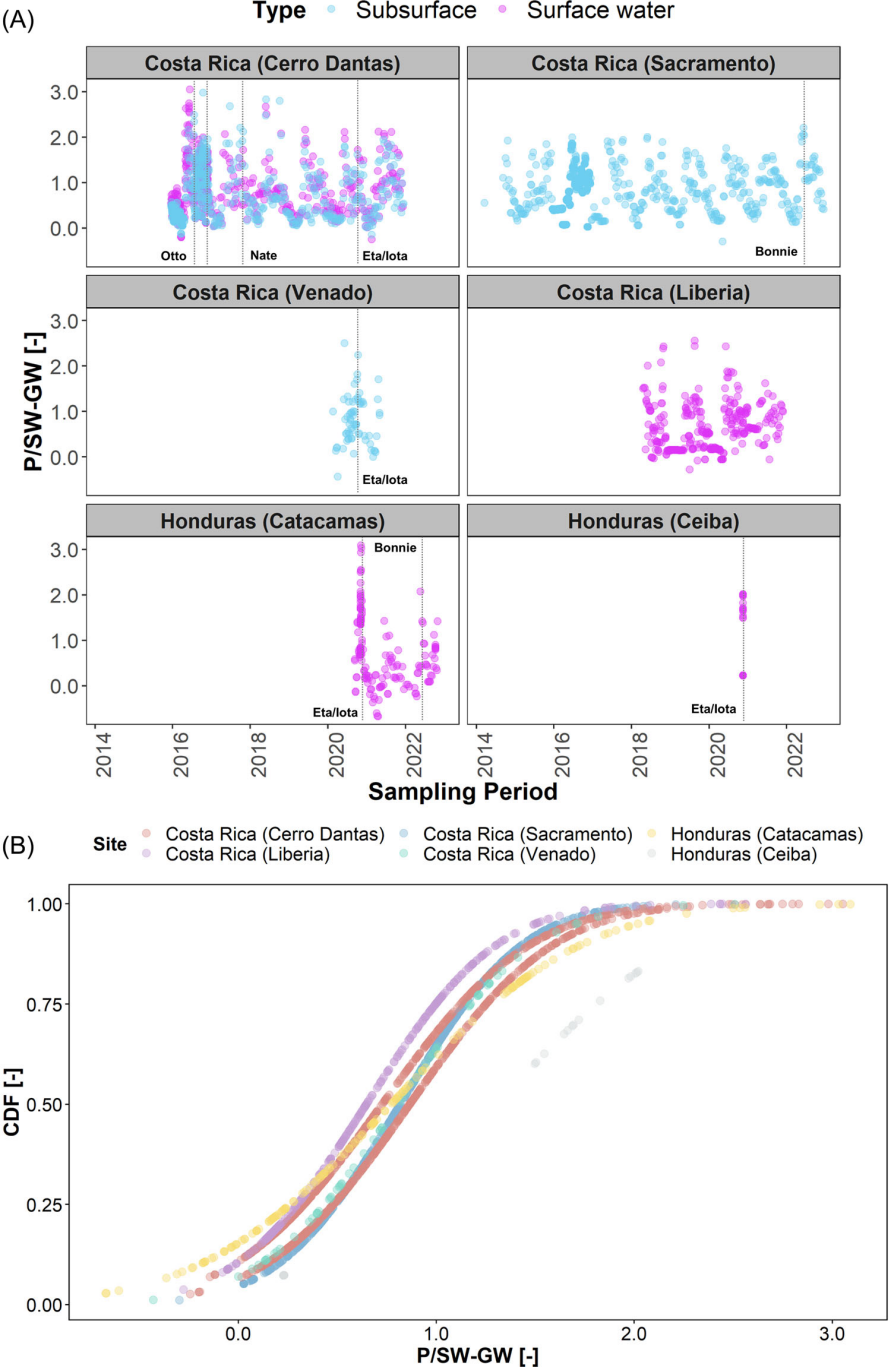
(A) Surface (pink) and subsurface (blue) daily precipitation (P)/surface water (SW) and P/groundwater (GW) [−] time series across Costa Rica and Honduras. Vertical dashed lines denote ratio spikes corresponding to tropical cyclones (TCs) isotopic excursions during Otto (2016), Nate (2017, Eta (2020), Iota (2020), and Bonnie (2022). (B) Cumulative distribution function (CDF) curves based on the P/SW and P/GW [−].

Figure 10 shows the time series of P/SW and P/GW [−] isotope ratios and their respective CDF curves. Seasonal isotope oscillations were depicted across SW and GW reservoirs. Greater P/SW and P/GW [−] ratios also occurred during the onset of the rainy season (May–June), when large isotope differences were commonly observed between the dry and wet seasons (up to ∼15‰). In Costa Rica, the influence of several TCs was recorded in the isotope spikes or increasing P/SW and P/GW [−] ratios: Otto (2016), Nate (2017), Eta/Iota (2020), and Bonnie (2022). However, despite the wet season onset signals, no indication of such isotope spikes was evident in the leeward Tempisque River (Liberia, Pacific slope). In Honduras, the influence of Eta (2020), Iota (2020), and Bonnie (2022) was recorded in similar high ratios. In general, these water bodies across Costa Rica and Honduras showed a large similarity in their hydrological response, with a cumulative probability close to 75% for P/SW and P/GW [−] ratios <1, except for El Cajón Dam (Honduras) releases during Eta (2020) and Iota (2020).

## DISCUSSION

This study presents the first comprehensive synthesis of water‐stable isotope compositions of 40 TCs across the North Atlantic, Gulf of Mexico, Caribbean Sea, and the Eastern Pacific Ocean basins, using isotope archives from 1984 to 1995 and more recent high‐frequency isotope data from 2012 to 2023. Previous regional efforts within the Intra Americas Sea^85^ and the Eastern Pacific have been focused on a single TC system or storms during a particular water year.^30^, ^31^, ^32^, ^45^, ^49^, ^50^, ^64^, ^65^, ^66^, ^86^, ^87^ Similar individual TC studies have been conducted in the Indian Ocean, Bay of Bengal, South China Sea,^51^, ^88^, ^89^, ^90^ and the western tropical Pacific Ocean.^91^, ^92^, ^93^, ^94^, ^95^ However, concerted tempestological networks (such as STORM) for monitoring water‐stable isotopes in vapor and discrete rain, SW, and GW samples in TC‐affected regions are extremely rare but urgently needed to better understand the spatiotemporal characteristics of TC‐associated rainfall, its contributions to the water budget, and importance for SW and GW reservoirs.

We tested and evaluated the three main underlying assumptions of the isotope storm spike hypothesis^45^, ^50^: (a) TC results in low isotope values, (b) TC isotope excursions propagate rapidly across freshwater environments, and (c) modern TC isotope ratios may be used for paleotempestological reconstructions. First, mean δ^18^O compositions across all TCs domains were significantly lower than the regional (noncyclonic) δ^18^O mean value (−5.24 ± 4.27‰): maritime (−6.29 ± 3.28‰), coastal (−7.78 ± 4.28‰), and inland (−9.80 ± 5.18‰; see *p* values in Figure 5B). However, low values (∼−17‰ to −22‰ in δ^18^O) accompanied by large rainfall events mainly occurred inland (e.g., central mountain ranges of Central America and Mexico), where orographic interaction plays a remarkable role in facilitating strong distillation and rainout.^96^ Notably, coastal and maritime TC convection resulted in large rainfall amounts with high isotope compositions. This is relevant since most terrestrial paleoproxies in Mesoamerica and the Intra Americas Sea are in coastal or maritime regions.^97^, ^98^, ^99^ The latter may bias paleoreconstructions in favor of heavy isotopes, commonly linked to drier conditions, particularly when analyzing annually resolved proxies. In addition, several TCs sampled at higher resolutions (e.g., Otto, Eta, Iota, Elsa, Isa, and Agatha) revealed a large isotope spectrum covering the entire annual range in δ^18^O and *d*‐excess. Linking high and low proxy compositions to drier (warmer) and wetter (cooler) based on the classical “amount effect”^52^ could be misleading when not considering the effects of active TCs seasons.

Second, P/SW and P/GW [−] isotope ratios revealed the rapid propagation of TCs isotope excursions in SW and GW systems. While this dataset is limited to seven sites in Costa Rica and Honduras, it is still unique as it provides high‐resolution measurements from single events to multiyear daily and weekly observations in the context of TC investigations. Increasing ratios indicated the dominant contribution of TC‐derived rainfall, reflecting a clear deviation from baseflow (prestorm) isotope compositions. While only one SW site was sampled from the Pacific leeward domain (Tempisque River, Liberia, Costa Rica), the P/SW indicator was only able to detect the onset of the rainy season (May to June), highlighting a potential limitation across the Pacific slope. In the windward sites, mainly located in the Caribbean slope or central mountainous regions, P/SW and P/GW ratios detected the TC excursions of Otto, Nate, Eta, Iota, and Bonnie. Similarly, the onset of the rainy season was also detected in the Caribbean domain. The onset of the rainy season (May–June) across Mesoamerica and the Intra Americas Sea resulted in low values (up to ∼−15‰ in δ^18^O).^31^ During the cold front (November–March) and dry season (December–April), sporadic rainfall events are characterized by high compositions (∼−3.0‰ to +5.0‰ in δ^18^O) because of small rainfall events and a predominant moisture source from the Caribbean Sea and Gulf of Mexico basins.^100^ Therefore, the occurrence of easterly tropical waves and the influence of the Intertropical Convergence Zone at the beginning of the rainy season produced low isotope values, resulting in similar trends in the P/SW and P/GW ratios.

Third, our results from 40 TCs suggest that the significant δ^18^O differences between TC‐derived and regional (noncyclonic) rainfall can be used to identify paleoepisodes of active cyclonic activity in inland territories.^32^, ^51^, ^91^ However, TC signals could also overlap with active rainy seasons, typically linked to La Niña years^101^ in the Mesoamerica and Caribbean regions. In addition, interpreting TC activity in coastal and maritime proxies could be problematic due to the occurrence of large rainfall events exhibiting high compositions, contrary to the classical “amount effect” paradigm.^52^

Regarding storm intensity, significant δ^18^O and *d*‐excess differences were found between hurricane categories (H1 to H5) and early or poststorm systems (LP, TD, and TS; see *p* values in Figure 8). H1, H3, and H4 events revealed similar δ^18^O and *d*‐excess compositions. The relatively high and low mean δ^18^O values in H5 and H2 events could be explained by their relatively low sample size and sampling location, concentrated in the maritime domains of the Caribbean Sea, Gulf of Mexico, and Atlantic Ocean (Irma, Dorian, and Ian) and the Pacific slope (Agatha), respectively. Category H1 and LP, TD, and TS events were not significantly different in δ^18^O median values. These results highlight the potential for assessing not only past wetter periods but also linking isotope compositions to the type of storm.

A remarkable feature is that hurricane events exhibited consistent spikes in lower *d*‐excess values than LP, TD, and TS. H2 and H5 exhibited mean *d*‐excess lower than +10‰, whereas H1, H3, and H4 categories showed greater *d*‐excess mean values. A detailed TC anatomy of Hurricane Otto (2016) revealed that increasing relative humidity and wind speed as the system organized toward its maximum category resulted in low *d*‐excess values up to −9.40‰, with high values up to +22.50‰ due to potential terrestrial moisture recycling.^31^ Similar low *d*‐excess observations have been reported during typhoon Shanshan (2006).^95^ However, other studies^32^, ^51^ have reported a significant negative correlation between δ^18^O and *d*‐excess compositions, whereas other studies reported diverging *d*‐excess values during Ita (2014; northern Australia) and across the metropolitan region of Manila, Philippines, respectively.^90^, ^91^ Certainly, as our study demonstrates, increasing the number of TC isotope observations across different domains and storm categories is needed to further assess the suitability of isotope compositions and paleostorm intensity.

Our results provide robust, spatiotemporal evidence of previously unknown water isotope variability in precipitation across one of the most active TC regions. Significant isotopic differences between maritime, coastal, and inland domains and within storm categories reinforce that paleoproxies analysis should not be based only on the classical “amount effect” paradigm, particularly in coastal regions, where large TC‐induced rainfall with high isotope values is a common modern feature. Freshwater isotope compositions support the assumption of rapid TC isotope excursions in SW and GW, but P/SW and P/GW ratios could overlap with the annual onset of active rainy seasons. Finally, the isotopic information confirms the highly active period between 2012 and 2023 in terms of TC frequency and landfalls over the study region, as revealed by our estimates of TC contributions to annual precipitation.

## CONCLUSIONS

The STORM network, combined with archived observations, provided a robust isotope baseline across 40 TCs in a large spectrum of maritime, terrestrial, and freshwater settings to evaluate the tropical storm spike hypothesis.^45^ Overall, our results reveal lower isotope compositions in maritime, coastal, and inland domains compared to regional (noncyclonic) long‐term rainfall, which is consistent with our TC‐contribution estimates. This highlights the potential of modern TC isotope monitoring for detecting paleo‐TC activity under different climate scenarios. For example, inland δ^18^O rainfall compositions were about 4‰ lower compared to the regional (noncyclonic) rainfall data. TC rainfall pulses were clearly damped (*D* ratios ranged from 0.12 to 0.43) throughout the study watersheds (Costa Rica and Honduras), but P/SW and P/GW ratios showed the rapid propagation of TCs in SW and GW as well as the onset of the rainy season, mainly in windward sites. However, despite the wet season onset signals, no indication of such isotope spikes was evident in the leeward study site. Significant δ^18^O and *d*‐excess differences were found between hurricane categories (H1 to H5) and early‐ or poststorm systems (low pressure, tropical depressions, and storms). These findings emphasize the value of modern ground TCs isotope observations for diagnosing storm intensity and frequency in paleoproxies.^51^, ^102^

The STORM network has greatly increased the capacity to integrate processes and interactions among the compartments of the water cycle beyond idealized models and, thus, has contributed to advances in the understanding of the short‐term scale impacts of heavy rainfall in freshwater systems that are often hindered by data scarcity at higher resolutions. Future networking activities include (a) the standardization of sampling protocols for tropical tempestology, (b) simultaneous vapor and discrete rainfall sampling, (c) sampling across different domains and storm categories, (d) the consolidation of an open‐access database, (e) data assimilation in paleoclimatic models, and (f) providing training and fostering collaborations with more research groups from the southern US Gulf Coast, the Caribbean region (Lesser and Greater Antilles), and Central America.

## AUTHOR CONTRIBUTIONS

Ricardo Sánchez‐Murillo conceptualized the monitoring network idea. Ricardo Sánchez‐Murillo, Kegan K. Farrick, Germain Esquivel‐Hernández, Rolando Sánchez‐Gutiérrez, Javier Barberena‐Moncada, Jorge Guatemala‐Herrera, Laura Gil‐Urrutia, Jorge Cardona‐Hernández, Junior O. Hernández‐Ortiz, Wendy Harrison‐Smith, Aurel Persiou, Juan Pérez‐Quezadas, Miguel Mejía‐González, Alejandro García‐Moya, Kristen Welsh, Rene M. Price, Diego A. Riveros‐Iregui, Minerva Sánchez‐Llull, Saúl Santos‐García, Geoffrey Marshall, and Adrián F. Obando‐Amador performed data collection and isotopic analysis. Dimitris A. Herrera provided the climatological data analysis. The first draft of the manuscript was written by Ricardo Sánchez‐Murillo and Dimitris A. Herrera. All the authors contributed and approved the final manuscript.

## CONFLICT OF INTEREST STATEMENT

The authors declare no conflicts of interest.

### PEER REVIEW

The peer review history for this article is available at: https://publons.com/publon/10.1111/nyas.15274

## Supporting information




**Figure S1** Best TC tracks for archived storm samples (1985–1995) based on the tropical cyclone historical database known as HURDAT2.^67,68^
**Figure S2** Best TC tracks for recent storms sampled across the tropical Pacific coast of Mexico based on the tropical cyclone historical database known as NC/NE HURDAT2.^67,68^
**Figure S3** Best TC tracks for recent (2013–2023) storms sampled across the Atlantic, Caribbean Sea, and Gulf of Mexico basins based on the tropical cyclone historical database known as HURDAT2.^67,68^

Supporting Information

Supporting Information

Supporting Information
