# Supplementary material for: Stable isotope tempestology of tropical cyclones across the North Atlantic and Eastern Pacific Ocean basins
Source: Ann N Y Acad Sci. 2024 Dec 19;1543(1):145–65. doi: 10.1111/nyas.15274 (PMC11776453; doi:10.1111/nyas.15274)
Supplement: Supplementary file 1 — Figure S1 Best TC tracks for archived storm samples (1985–1995) based on the tropical cyclone historical database known as HURDAT2.67,68 Figure S2 Best TC tracks for recent storms sampled across the tropical Pacific coast of Mexico based on the tropical cyclone historical database known as NC/NE HURDAT2.67,68 Figure S3 Best TC tracks for recent (2013–2023) storms sampled across the Atlantic, Caribbean Sea, and Gulf of Mexico basins based on the tropical cyclone historical database known as HURDAT2.67,68 [file NYAS-1543-145-s002.docx]

**
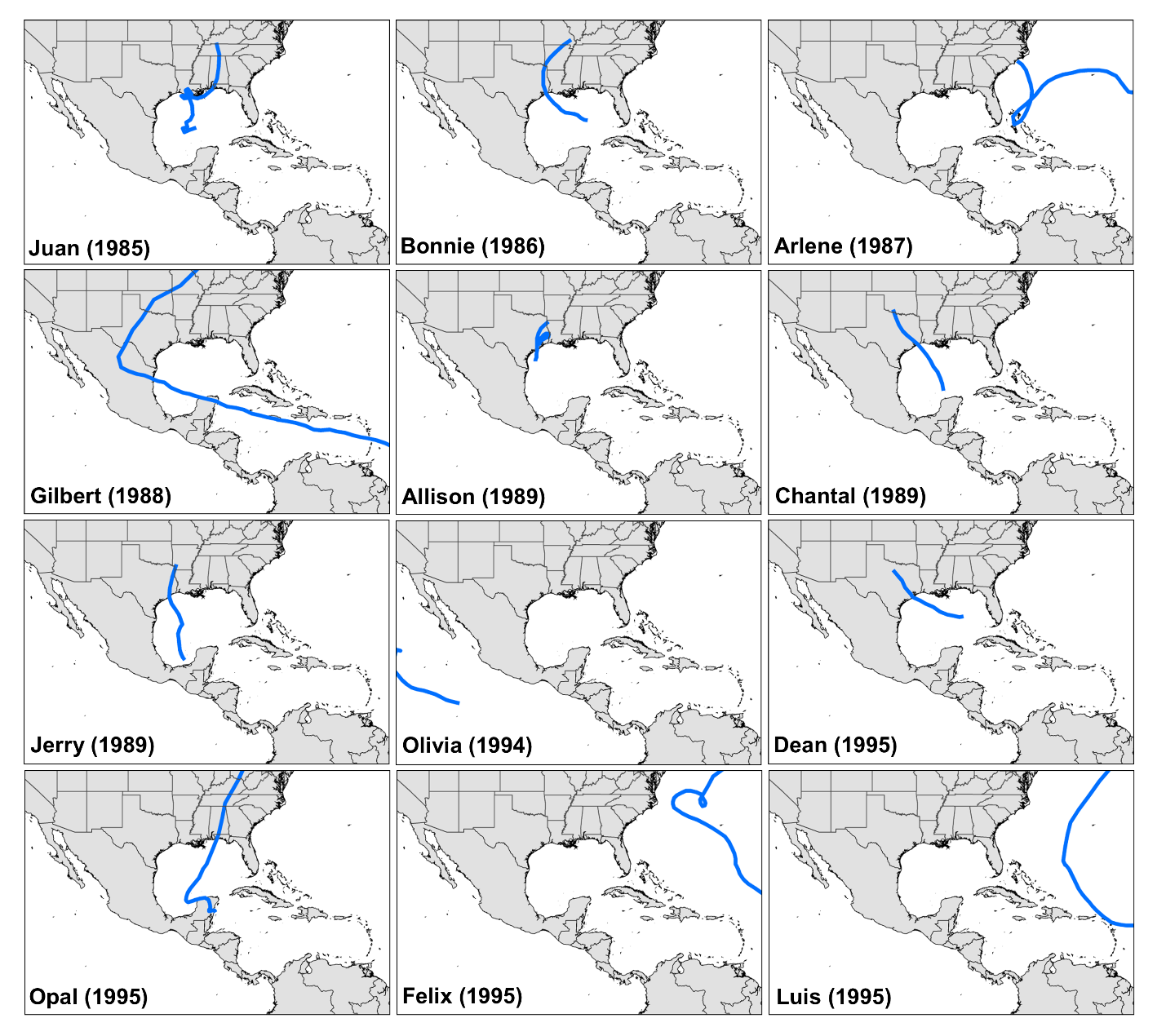
**

**Figure S1:** Best TC tracks for archived storm samples (1985-1995) based on the tropical cyclone historical database known as HURDAT2^67-68^.

**
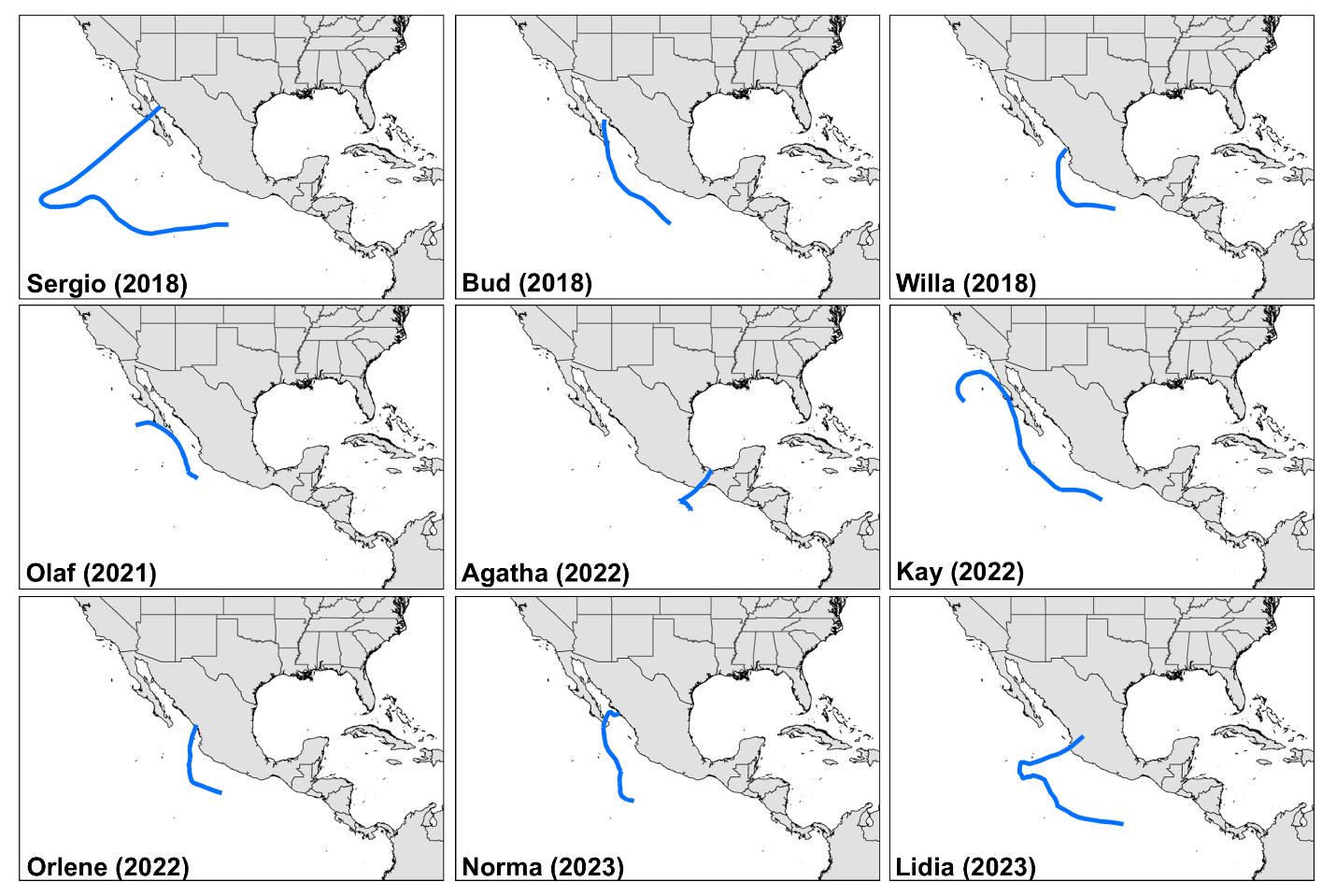
**

**Figure S2:** Best TC tracks for recent storms sampled across the tropical Pacific coast of Mexico based on the tropical cyclone historical database known as NC/NE HURDAT2^67-68^.


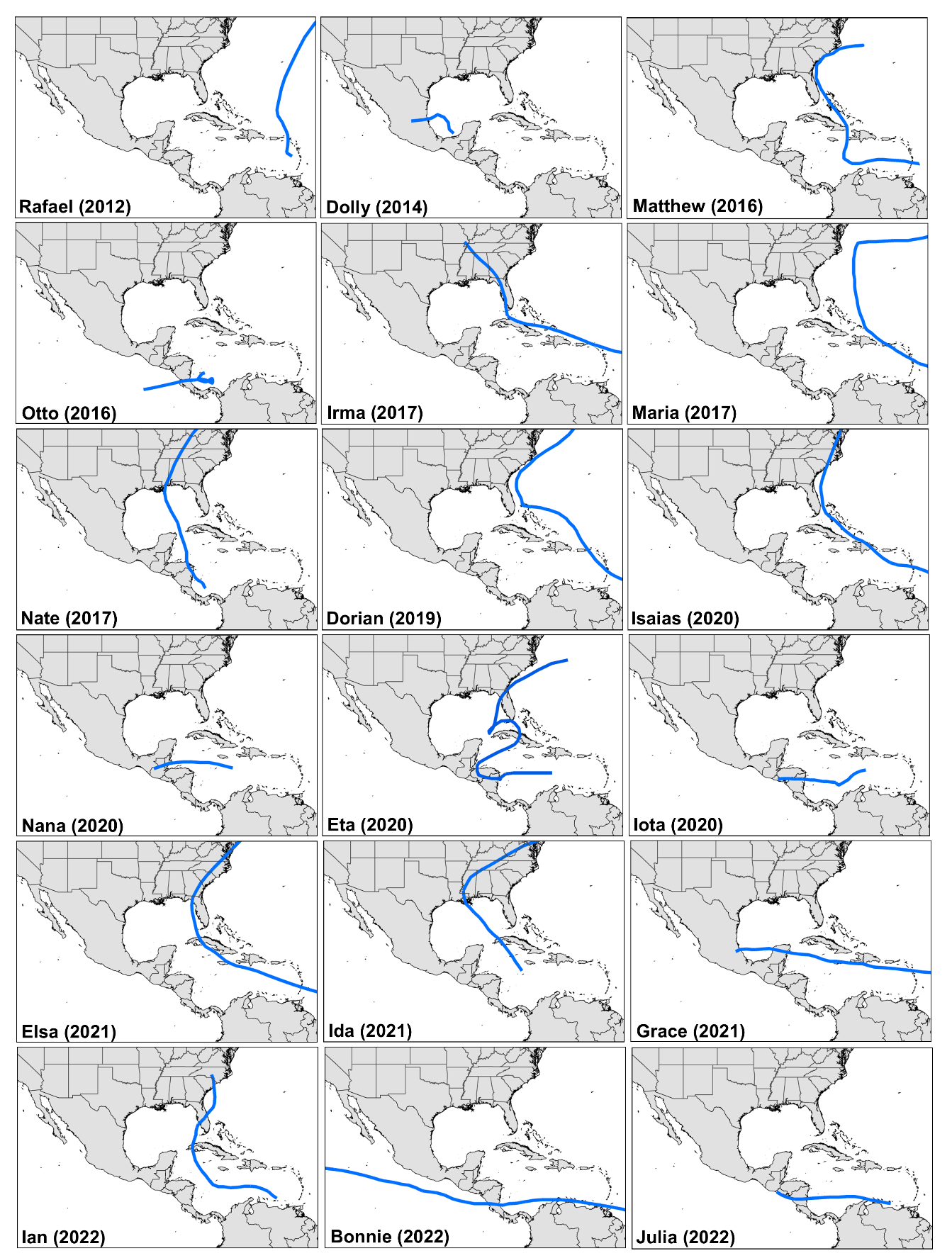


**Figure S3:** Best TC tracks for recent (2013-2023) storms sampled across the Atlantic, Caribbean Sea, and Gulf of Mexico basins based on the tropical cyclone historical database known as HURDAT2^67-68^.
